# Inadequate timing of prophylactic antibiotics in orthopedic surgery. We can do better

**DOI:** 10.3109/17453670903316868

**Published:** 2009-12-04

**Authors:** Anna Stefánsdóttir, Otto Robertsson, Annette W-Dahl, Sverrir Kiernan, Pelle Gustafson, Lars Lidgren

**Affiliations:** ^1^Department of Orthopedics and Clinical Sciences, Lund University Hospital, Lund, Sweden; ^2^WHO Collaborating Centre for Evidence-Based Health Care in Musculoskeletal Disorders

## Abstract

**Background and purpose** There are rising concerns about the frequency of infection after arthroplasty surgery. Prophylactic antibiotics are an important part of the preventive measures. As their effect is related to the timing of administration, it is important to follow how the routines with preoperative prophylactic antibiotics are working.

**Methods** In 114 consecutive cases treated at our own university clinic in Lund during 2008, the time of administration of preoperative prophylactic antibiotic in relation to the start of surgery was recorded from a computerized operation report. In 291 other cases of primary total knee arthroplasty (TKA), randomly selected from the Swedish Knee Arthroplasty Register (SKAR), the type and dose of prophylactic antibiotic as well as the time of administration in relation to the inflation of a tourniquet and to the start of surgery was recorded from anesthetic records.

**Results** 45% (95% CI: 36–54) of the patients operated in Lund and 57% (CI: 50–64) of the TKAs randomly selected from the SKAR received the preoperative antibiotic 15–45 min before the start of surgery. 53% (CI: 46–61) received antibiotics 15–45 min before inflation of a tourniquet.

**Interpretation** The inadequate timing of prophylactic antibiotics indicates that the standards of strict antiseptic and aseptic routines in arthroplasty surgery are falling. The use of a simple checklist to ensure the surgical safety may be one way of reducing infections in arthroplasty surgery.

## Introduction

Today, there is increasing concern that the number of infections in conjunction with arthroplasty surgery is slowly rising. At a recent meeting of the Nordic Arthroplasty Association (NARA), Denmark, Norway, and Sweden indicated an increase in infections after hip arthroplasty after the year 2000. Although a similar trend has not been observed for knee arthroplasty, it must be kept in mind that the risk of knee infection is already higher. The figures from the Nordic arthroplasty registers (http://www.jru.orthop.gu.se, http://www.knee.se, http://www.dhr.dk/english.htm, http://www.haukeland.no/nrl/eng) are well in accordance with a recent study by Pulido et al. (2008) who reported an infection rate after hip and knee arthroplasty of 0.3% (15 of 5,060) and 1.1% (48 of 4,185), respectively (p < 0.001).

When arthroplasty was introduced in the Nordic countries, it was a highly specialized type of surgery with strict antiseptic and aseptic routines. There was meticulous attention paid to antibiotic prophylaxis, outpatient scrubbing (repeated by the hospital staff before and at surgery), and checking for skin infections and other ongoing infections—and the patient was to be admitted to a clean ward, or at least to a clean single room, as late as possible before the actual surgery (Lidgren and Rasmusson 1973).

There are indications that the standards have slowly fallen. Patients are now often admitted to general wards together with other elective and emergency cases, and the wards are often overcrowded. This is worrying, considering that there is clear evidence that the risk of hospital-acquired infection and bacterial resistance increases when bed occupancy is above 90% (Marx 2008). Furthermore, arthroplasty surgery is now performed as routine surgery, often by staff with varying levels of experience. This “industrialization” has probably made it increasingly difficult to constantly maintain important prophylactic routines.

Antibiotic prophylaxis is important to minimize the risk of infection, and the evidence for its use has been growing. A recent systematic review (AlBuhairan et al. 2008) of the effectiveness of antibiotic prophylaxis in patients undergoing total hip and knee replacement found that antibiotic prophylaxis reduced the absolute risk of wound infection by 81% compared with no prophylaxis (p < 0.001) (pooled analysis of 7 studies; n = 3,065). Furthermore, a study from the Norwegian Arthroplasty Register on total hip arthroplasties found that combining systemic antibiotics with antibiotic-impregnated cement significantly reduced the risk of infection and that the combination was superior to the single use of either of these prophylactic methods (Engesaeter et al. 2003).

To ensure that there is an adequate antibiotic concentration in the tissues at surgery, the timing of preoperative prophylaxis is crucial. For the most commonly used antibiotics, it has been considered optimal to administer the drug intravenously 30 min before skin incision (Gyssens 1999, Polk and Christmas 2000) and it has been documented that administration more than 60 min preoperatively is associated with higher risk of surgical infection (Galandiuk et al. 1989).

The importance of antibiotic prophylaxis and its timing is illustrated by the fact that in the US, experts in surgical infection prevention, hospital infection control, and epidemiology have developed guidelines for national surveillance and quality improvement in giving prophylaxis (Bratzler and Houck 2004).

In a recent study of 1,922 consecutive hip arthroplasty patients from 11 hospitals, it was found that surgical site infection (superficial and deep) occurred in 2.6% of cases. The highest odds for infection were found in patients who had received prophylaxis after incision, and the authors suggested that intervention programs in search of amendable factors to prevent infection should focus on timely administration of antibiotic prophylaxis (van Kasteren et al. 2007).

A small study at our own university clinic in Lund, initiated by a local strategic program against antibiotic resistance, indicated that the timing of prophylactic antibiotics was inadequate. We decided to study the timing of administration of prophylactic antibiotics at our own clinic in greater detail. We also examined a sample of knee arthroplasties reported to the Swedish Knee Arthroplasty Register (SKAR).

## Patients and methods

In 114 consecutive cases treated at our own university clinic in Lund during 2008, the time of administration of preoperative prophylactic antibiotic in relation to the start of surgery was recorded from the computerized operation report. The information was collected without the involvement or knowledge of the staff who administered the prophylactic antibiotic. According to local guidelines, patients should have the preoperative prophylactic antibiotic 30 min before the start of surgery. The time of inflation of a tourniquet was not available.

The SKAR contains a restricted number of variables (Robertsson et al. 2000), and the timing of prophylactic antibiotics was not registered before 2009. To search for this information, 300 cases were randomly selected from the 9,238 primary total knee arthroplasties (TKAs) registered in SKAR to have been performed due to osteoarthritis during 2007. A random-number generator was used. The anesthetic report was requested from the operating unit and searched for information on the type and dose of prophylactic antibiotic, as well as the time of administration in relation to the inflation of a tourniquet and to the start of surgery.

The anesthetic records were unavailable for 9 patients (3%), leaving 115 men and 176 women. The mean age at operation was 70 (43–90) years. 4 patients had both knees operated on the same day and in 3 cases the knee selected for study was the first one and in 1 the second. Administration of prophylactic antibiotic more than 45 min before the start of surgery was regarded as inadequate because of the short half-life of the most commonly used antibiotics. Administration later than 15 min before the start of surgery was also regarded as inadequate, as in most cases the infusion will not have entered the circulation at the time of incision; it is definitely too late when a tourniquet is used (Tomita and Motokawa 2007).

The 95% confidence interval (CI) for proportion was calculated as p_s_ ± 1.96 times the standard error of p_s_, where p_s_ is the proportion of patients receiving prophylactic antibiotic in adequate time.

## Results

**Figure 1.**
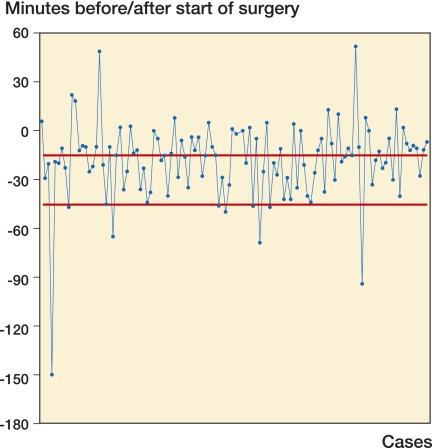
A control chart showing the timing of the first dose of prophylactic antibiotic in relation to the start of surgery at the Department of Orthopedics, Lund University Hospital. Each dot represents 1 case. Zero represents the start of surgery. The red lines indicate 45 min and 15 min before the start of surgery.

Of the 114 patients studied in Lund during 2008, only 51 (45%, 95% CI: 36–54%) received the first antibiotic dose 15–45 min before the start of surgery (Figure 1). In 22 cases (19%), surgery was started at the same time or before administration of prophylactic antibiotic.

**Figure 2.**
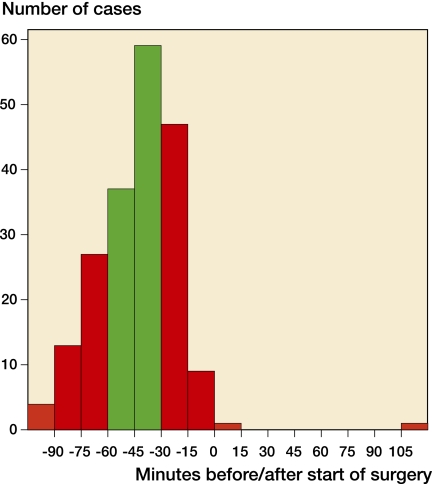
The timing of administration of prophylactic antibiotic in relation to the start of surgery in 198 cases of primary TKA. Zero represents the start of surgery. The green bars correspond to acceptable timing.

**Figure 3.**
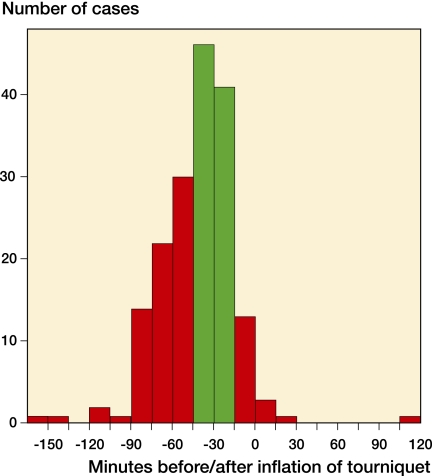
The timing of administration of prophylactic antibiotic in relation to the inflation of a tourniquet in 176 cases of primary TKA. Zero represents the start of surgery. The green bars correspond to acceptable timing.

Of the 291 randomly selected TKAs from the Swedish Knee Arthroplasty Register, it was possible to get information on the type of prophylactic antibiotic administered in 247 cases (85%). 89% had received cloxacillin. The dose varied and there seemed to be no common standard (Table). In 198 cases (68%), it was possible to ascertain from the anesthetic record the time from administration of the prophylactic antibiotic until the start of surgery. Only 113 of 198 (57%, CI: 50–64%) received the antibiotic 15–45 min before the start of surgery (Figure 2). The mean time was 41 min, with a range from 105 min before the start of operation to 120 min after the start. In 176 cases (61%), it was possible to ascertain the time from administration of prophylactic antibiotic until the time of inflation of a tourniquet. Only 94 of 176 patients (53%, CI: 46–61%) received antibiotics 15–45 min before the tourniquet was applied (Figure 3). The mean time was 40 min, with a range from 153 min before the inflation of a tourniquet to 120 min after inflation.

**Table:** The intravenous antibiotic prophylaxis administered preoperatively in 247 randomly chosen TKAs performed in Sweden 2007

Antibiotic	n	%
Cloxacillin		
1 g	54	22
2 g	158	64
unknown dose	7	3
Clindamycin		
300 mg	1	0.4
600 mg	23	9
Cefuroxime		
1.5 g	4	2

In 2 of the 4 bilaterally operated patients, no additional antibiotics were given before the start of surgery on the second knee.

## Discussion

There is good evidence for the use of prophylactic antibiotics to reduce the risk of surgical wound infection (Classen et al. 1992, Lidgren 2001, Fletcher et al. 2007, Prokuski 2008) and for how preoperative antibiotic prophylaxis should be administered. The adherence to recommendations is, however, not well studied. In our clinic at Lund University Hospital, we found a lack of adherence to prophylactic routines with less than 50% of cases receiving antibiotics 15–45 min before the start of surgery. The national sample collected from the Swedish Knee Arthroplasty Register showed the same disturbing result: only 57% had adequate timing of antibiotic prophylaxis. 2 of 4 bilaterally operated patients did not get a second antibiotic infusion before the second knee arthroplasty. This alarming result raises concern about the standard of other established measures to reduce the risk of infection.

It was recently shown in a non-randomized study that use of a simple surgical safety checklist reduced morbidity and mortality (Haynes et al. 2009). Interestingly, the surgical site infection rate was reduced by almost 50% (p < 0.001). The administration of antibiotics within 60 min before incision improved from 56% to 83% by use of the safety list.

There are indications from the national registers in Denmark, Norway, and Sweden that infection rates after primary hip arthroplasty are increasing. Furthermore, the proportion of revisions caused by infection is increasing (http://www.jru.orthop.gu.se, http://www.dhr.dk/english.htm, http://www.haukeland.no/nrl/default.htm). The reoperation rate because of infection within 2 years after a primary hip arthroplasty for those operated in Sweden 2004–2007 was 0.6%, and there was an unacceptable 10-fold difference when comparing individual surgical units in Sweden, with a range of 0.2–2.0% (http://www.jru.orthop.gu). The true incidence of infection is higher, as some cases are treated with antibiotics only. Some low-virulence infections will lead to a slow, progressive loosening and the diagnosis of infection may sometimes be obvious first at revision with positive cultures often more than 2 years after surgery. The large differences between clinics can only be partially explained by differences in case mix such as sex, replacement for hip fracture, rheumatoid arthritis, and tumor, all of which have been reported to influence the risk of prosthetic infection (http://www.knee.se, Bengtson 1990, Berbari et al. 1998, Gjertsen et al. 2008).

Thus, the statement in an editorial of this journal in 2001 that “joint prosthetic infection is a success story” (Lidgren 2001) may not be true any longer. Apart from inadequate administration of prophylactic antibiotics, possible reasons might be overcrowded wards with mixed patients and lack of focus on environmental hygiene measures (Dancer 2008, Patel et al. 2008). It has been shown that a segregation policy with fenced wards results in a reduced infection rate in those undergoing elective joint replacement, and also reduces MRSA colonization (Biant et al. 2004, Johnston et al. 2005).

The first study published on prophylactic antibiotics in joint replacements came from Sweden (Ericson et al. 1973) and ever since, cloxacillin has been the prophylactic antibiotic most used in orthopedic surgery in Sweden. There is a lack of recent publications on local resistance patterns, based on preoperative screening of nasal carriers and on cultures at revision (Sanzén and Walder 1988, Stefánsdóttir et al. 2009), to decide whether the antibiotics in current use are effective or whether the prophylaxis should be changed.

A factor that is not normally taken into account is the relatively short half-life of cloxacillin (30 min). The half-life of cefuroxim is 66 min and that of clindamycin is 144 min. Earlier randomized studies on the effect of different regimes often used systemic administration of antibiotics either as intravenous injections or (in the 1970s) intramuscular injections. Today, a preoperative infusion of antibiotics is often given, which may last for up to 30 min although a shorter infusion time of 15 min is more common.

The prophylaxis has least effect when an antibiotic is given after the application of a tourniquet. This means that the extremity is to a large extent unprotected regarding antibiotic prophylaxis, because although local antibiotic-containing bone cement is used routinely in knee prostheses in the Nordic countries, the antibiotics will only start to leak out slowly from the outer surface of bone cement once the tourniquet has been released and bleeding starts (Törholm et al. 1983, Lidgren et al. 2003). Similarly, there is a considerable reduction in protection when a patient is given an antibiotic with a short half-life too early i.e. too long before the operation starts.

The antibiotic regimes typically recommend fixed doses of antibiotics pre- and postoperatively, with the first postoperative dose often being administered 6 h after surgery. It would probably be more efficient to individually prescribe a dose based on body weight and to repeat the dose based on the half-life of the antibiotics selected as well as the length of the surgical procedure. Thus, one could consider whether or not all patients for whom the actual surgical procedure takes more than 1 hour should have a new infusion, starting just before the tourniquet is released. This is also what the AAOS recommends in its recent document on infection prophylaxis (Prokuski 2008). The document states that at twice the half-life of the selected antibiotic (counting from the first injection), a repeat dose should be given. In knee arthroplasty surgery, this very often coincides with the release of the tourniquet. One should be aware of the fact that the half-life given, for example, for cloxacillin (30 min) is based on studies in healthy individuals and microbiology assays. Hepatic biotransformation may also induce a slight risk of accumulation of penicillins. Furthermore, it is known that patients undergoing hip and knee arthroplasty develop a transient globular and tubular dysfunction. However, systemic prophylaxis with cloxacillin and bone cement containing gentamycin has not been considered to be the cause of this, but rather the surgical trauma (Nergelius et al. 1997a,b, 1998, Vinge et al. 1997).

One way of circumventing some of the difficulties of timing in knee surgery would be to use regional antibiotic treatment by local intravenous administration in the foot, given prior to surgery but after tourniquet application. This has been shown to give up to 8 times the tissue concentration compared to systemic administration, but there are no published data on the feasibility of adapting it widely in clinical practice (Miller et al. 2004). Another way might be to use continuous antibiotic infusion during the whole procedure, starting 45 min before surgery, and to calculate the speed of infusion (i.e. the amount given) based on well-known information such as the serum and tissue profile of the prophylactic antibiotic(s) selected, and also the age of the patient, their kidney function and BMI, and adjust accordingly (Amendola 2000). This would keep the patient protected in the case of a hip arthroplasty but would only partly solve the problem for knee arthroplasty, as the use of a tourniquet is standard today.

In a recent study by Sorian et al. (2008), 900 knee arthroplasty patients were randomized between standard antibiotic prophylaxis and administration of prophylactic antibiotics just before release of the tourniquet. In both the standard and the experimental group, the patients had a high rate of deep infection at three months of follow-up (of 3.4% and 1.9%, respectively), but the difference was not statistically significant. That particular study raises serious concern about the present timing of administration of antibiotic prophylaxis in knee arthroplasty.

### Clinical relevance

Our findings show that the timing of preoperative antibiotic prophylaxis is inadequate and that the routines must be sharpened. Furthermore, it seems that the literature supports adjustment of doses, timing of injections, and also repeated injections during lengthy surgical procedures based on the half-life of the antibiotic(s) selected. We suggest that orthopedic clinics should start to use a simple checklist for surgery with a standard delay before skin incision, during which (among other important considerations) the administration of prophylactic antibiotics is confirmed. As of January 2009, the time of administration of preoperative antibiotic has been recorded in the Swedish Knee Arthroplasty Register.
